# Changing organizational culture in community sport: a systematic review

**DOI:** 10.3389/fspor.2026.1852179

**Published:** 2026-06-15

**Authors:** James Woodforde, George Thomas, Zoe Harrison, Samantha Mulcahy, Stephen Townsend, John Cairney

**Affiliations:** 1 School of Human Movement and Nutrition Sciences, Faculty of Health, Medicine and Behavioural Sciences, The University of Queensland, Brisbane, QLD, Australia; 2 Health and Wellbeing Centre for Research Innovation, Brisbane, QLD, Australia; 3 The Queensland Centre for Olympic and Paralympic Studies, Brisbane, QLD, Australia

**Keywords:** community sport, organizational culture, review, social inclusion, sport management

## Abstract

**Introduction:**

Community sport organizations – encompassing grassroots, recreational, amateur and voluntary clubs and settings distinct from elite or professional sport – are increasingly positioned as sites for advancing social inclusion, health and wellbeing, yet the role of organizational culture in enabling or constraining these outcomes is not fully understood. While policy and practice increasingly call for culture change in community sport, the extent to which such efforts have been empirically evaluated remains unknown.

**Methods:**

A systematic review was conducted across five databases (PubMed, SPORTDiscus, PsycINFO, Web of Science, Scopus) from inception to June 2025. Studies were included if they evaluated an intentional strategy described as aiming to change culture in community sport settings and reported participant- or organizational-level outcomes. During title/abstract screening by reviewers, records were prioritized using machine-learning algorithms to rank relevance. Study quality was appraised using the Mixed Methods Appraisal Tool.

**Results:**

Twelve studies met inclusion criteria. Most were qualitative, small-scale, and context-specific. Culture change efforts clustered thematically around: (i) integrity and safety cultures, (ii) inclusion and diversity for marginalized groups, and (iii) adult conduct in youth sport settings and coach development. While the evidence base meeting our criteria is nascent and heterogeneous, precluding quantitative synthesis, findings suggest that deliberate, multi-level culture change efforts can produce positive shifts in attitudes, behaviors and club practices, particularly when initiatives combine individual learning with organizational-level change and sustained external support.

**Discussion:**

Culture change in community sport settings is complex, uneven and often gradual, influenced by entrenched traditions and resourcing constraints. Future efforts should prioritize longitudinal evaluation designs, clearer conceptual and operational definitions of culture change, and theoretically grounded frameworks that can be compared across contexts.

## Introduction

Community sport is one of the most widely accessed and socially embedded forms of leisure-time physical activity (1). Its contributions to physical health, social connection and a sense of belonging for people across the lifespan are well known (2–4). Typically characterized by local organization, volunteer involvement and an emphasis on participation rather than elite performance, community sport organizations (CSOs) play a central role in providing accessible sport opportunities within communities (5). As such, they are increasingly recognized not only as sites for physical activity, but also as important social institutions through which broader outcomes related to inclusion, wellbeing and social cohesion may be realized (6).

While the benefits of participating in community sport are well documented, these outcomes are not guaranteed and are not experienced by all members of society. Instead, they may be directly or indirectly influenced by the underlying culture of sporting environments, which can either support or constrain participation, enjoyment and continued engagement in sport. For example, a systematic review by Crossman et al. (7) identified sport culture as a key organizational-level factor in encouraging adult sport participation, particularly when environments were supportive, inclusive and accommodating of individual differences. In the context of social integration of disabled members, Albrecht et al. (8) similarly reported that a club's openness and atmosphere of welcome and equal acceptance were crucial. Evidence from youth sport further suggests that these social and environmental features are closely tied to participation (9). However, when club cultures are perceived as inflexible and unwelcoming, or demonstrate a lack of cultural awareness, they can act as barriers to continued participation (10, 11). In junior sport, Spaaij et al. (12) found that a strong performance focus and rigid gender norms were associated with reduced commitment to diversity and less inclusive attitudes. As Anderson (13) argues, such negative experiences are not inevitable but can result from a culture obsessed with winning and underpinned by deeply embedded structural practices.

Despite its widespread use in both academic and public discourse, the concept of culture as it relates to sport is not always clearly defined. In sporting settings, *culture* is often used informally in reference to the values, behaviors and norms that shape how teams or clubs operate (14), such as having a strong ‘team culture’ or challenging a ‘toxic culture’. In research, the concept of culture is applied across sociocultural analyses of sport that examine identity, power and meaning, as well as organizational studies concerned with how values and norms are produced and sustained within sport settings. While recognizing the overlap between these traditions, this review focuses on organizational culture as enacted within CSOs – that is, how broader social norms are embedded in, negotiated through, and potentially influenced by organizational practices, structures and routines. Organizational culture in sport has been studied using a range of paradigms, perspectives and definitions, with limited consensus on how it is conceptualized or operationalized (14). More recently, drawing on six foundational models from the management and organizational literature, Cairney et al. (15) proposed an integrative framework that synthesizes layered, typological and contextual dimensions of culture in sport. Identified in that work as one of the most influential frameworks is Schein's (16) model, which conceptualizes culture as comprising three inter-related levels: visible artefacts (e.g., rituals, routines), espoused values (e.g., mission statements), and underlying assumptions (deeply held beliefs and norms). Within this view, organizational culture can be understood as the collection of values, beliefs and attitudes that are shared among members of a sport organization and that shape how ‘things are done’ and what is considered acceptable (17). Accordingly, in this review, *culture change* is understood as intentional efforts to shift these shared norms, values and assumptions within CSOs, pursued through changes to organizational structures, practices, governance or member-behaviors.

In practice, policy has increasingly sought to influence organizational culture across all levels of sport. Across many countries, national sport strategies and sector initiatives promote values such as respect, inclusion, safety and integrity. In Australia, this is reflected in the National Sport Strategy (2024–2034) (18), alongside national programs such as the Good Sports initiative, which supports safer and more family-friendly club environments (19), and Club Respect, which helps clubs build and sustain “a deep culture of respect” (20). Similar priorities are evident in inclusion strategies developed by national sporting organizations – such as the Australian Football League's LGBTQI + Inclusion Action Plan (21) and the National Rugby League's Inclusion Framework (22) – as well as in initiatives like the Positive Coaching Alliance in the United States, which aims for all children to have access to a positive youth sport experience (23).

Despite this policy and practice emphasis on culture change in community sport, it remains unclear how CSOs are attempting to develop or change their organizational culture, and to what extent such efforts have been evaluated or shown to influence organizational or participant-level outcomes. Community sport organizations are embedded within broader systems of interconnected persons and contexts – including community networks, governance structures, policy environments and cultural norms – that shape and are shaped by what occurs within them (24, 25), and it is within this context that efforts to change organizational culture can be understood. A growing body of scholarship at the intersection of organizational psychology and sport examines how culture, leadership and organizational dynamics affect athlete experiences and performance outcomes (26, 27). This work has generated important insights into how cultures form, persist and can become destructive in elite sport settings (28), and has called for greater attention to the organizational conditions that enable both performance and wellbeing (29, 30). However, much of the existing research on organizational culture in sport has centered on elite, professional and high-performance settings (14), where formal structures, paid staff and performance goals differ significantly from the volunteer-driven, participation-focused environments of community sport which may pose unique organizational challenges (25). Even in reviews that include a broader span of sport organization types, the primary focus has been on how culture is defined, conceptualized and studied, rather than synthesizing literature on efforts to influence culture and their impacts (14, 27). Considering these gaps, this review aims to synthesize and examine evaluated efforts to change culture in community sport settings, and to assess the outcomes associated with those efforts.

## Methods

The protocol for this review was registered with the Open Science Framework (registration ID: 98WTE) in accordance with PRISMA-P guidelines (31).

### Inclusion criteria

Studies were eligible for inclusion if they focused on community sport conducted in grassroots, recreational, amateur or voluntary contexts, rather than elite, professional or high-performance sport. School-based sport was eligible only where it reflected participation-focused structures rather than elite pathways. Studies needed to evaluate an intentional strategy implemented within existing community sport structures that explicitly sought to influence, support or change culture. For the purposes of screening, culture change was operationalized as any intentional effort to shift shared values, norms, behaviors, or organizational structures within the community sport setting, where the original study authors explicitly framed the initiative as related to ‘culture’ or ‘cultural’ change in the title or abstract (reflected in our search strategy; see *Search Procedure*). This means inclusion was based on authors’ own framing of their initiative as culture-related, rather than on an independent determination that the intervention achieved or constituted genuine transformation of culture. Included studies were also required to report on outcomes or impacts of the strategy, using either qualitative or quantitative methods, in any domain (e.g., social, cultural, health, economic). Reports limited to implementation processes, without outcome data, were not eligible. Only peer-reviewed, original research articles published in English were considered.

Studies were excluded if they focused on curricular physical education in schools, described sport culture without an evaluative component, or used sport primarily as a vehicle for broader social outcomes (e.g., crime reduction, refugee integration, literacy) without addressing culture within the existing sport environment. Similarly, interventions targeting only individual or developmental outcomes (e.g., life skills, confidence, social skills, mental health) were excluded where they did not explicitly seek to shift shared values, norms, or practices of the sport setting itself.

### Search procedure

A systematic search was conducted across five academic databases (PubMed, SPORTDiscus, PsycINFO, Web of Science and Scopus) from inception to June 2025. The strategy was designed to be sufficiently broad to capture studies examining sport culture within the community context. Search terms spanned three domains connected using Boolean operators: (1) sport (e.g., *sport*, database-specific subject headings relating to specific sports); (2) setting descriptors (e.g., *community*, *grassroots*, *amateur*); and (3) *culture* or *cultural*. To maintain conceptual coherence, and consistent with the approach used by a similar systematic review (14), the search was limited to studies that explicitly used the term *culture* or *cultural* in the title or abstract. Terms relating to study outcomes were not included, as restricting by outcome has been shown to reduce retrieval of relevant records (32). Filters were applied to restrict results to English-language, peer-reviewed journal articles. The search strategy is presented in full in Supplementary Table S1.

### Screening and study selection

Following duplicate removal in EndNote X9.3.3, records retrieved from the database searches were imported into an open-source machine learning tool for prioritizing title and abstract screening (ASReview v1.6.5). Screening was informed by the SAFE procedure, a practical and conservative framework for determining stopping points in active learning–assisted systematic review screening (33). SAFE combines multiple heuristics, including preliminary training data, explicit stopping criteria, model switching and *post-hoc* verification steps to maintain transparency and minimize the risk of excluding relevant studies (33). The use of ASReview is supported by growing methodological evidence demonstrating that machine learning–assisted screening can substantially reduce screening burden while maintaining high recall of relevant studies when combined with human verification (34, 35).

Full details of the model configurations used across each screening phase are provided in Supplementary Table S2. Prior to machine learning–assisted screening, a purposive sample of 50 records was independently screened by five members of the research team. Discrepancies were resolved through discussion to achieve consensus, and the resulting inclusion decisions were used as high-confidence training labels to initialize the model in the active learning phase. Screening in ASReview entailed four phases each with predesignated stopping criteria, with one reviewer (JW) completing the active learning phase and a second reviewer (GT) conducting an additional verification phase. The stopping criteria for the main active learning phase required a minimum of 10% of the dataset screened (*n* ≥ 1,459), at least twice the estimated number of relevant records screened, and no additional relevant records found in the final 50 records screened. Screening concluded when final stopping rules were met after 1,510 records, yielding 116 records for full-text review.

Two reviewers (JW, ZH) independently conducted full-text screening, with discrepancies resolved involving a third reviewer (GT). Reference lists of the included studies were also searched, but no additional eligible studies were identified.

### Data extraction and synthesis

A structured extraction template was developed during protocol development and used to record key study characteristics relevant to the review aims. Extracted items included bibliographic details, study design, country, sport context, participant population, description of the culture change strategy or intervention, and reported outcomes. One reviewer (ZH) completed the initial extraction for all included studies, before a second reviewer (JW) cross-checked each entry for accuracy against the source material.

Extracted data were summarized in tabular form (Table 1) to provide an overview of study characteristics. Given the heterogeneity of designs, contexts and outcomes, a narrative synthesis approach was used. Studies were described individually to preserve detail and then grouped thematically in-text according to the primary focus of the culture change effort (e.g., alcohol culture, inclusion, youth sport values). This approach allowed integration across diverse methods while retaining the contextual nuance of each study.

**Table 1:** Summary of study characteristics.

Characteristic	Category	No. of studies	% of Total
Publication Year	2020–2024	6	50%
	2015–2019	5	42%
	2010–2014	1	8%
Country	Australia	5	42%
	Brazil	1	8%
	England	1	8%
	Germany	1	8%
	Greece	1	8%
	Italy	1	8%
	New Zealand	1	8%
	United States	1	8%
Study Design	Qualitative	7	58%
	Quantitative	3	25%
	Mixed methods	2	17%
Sport Level	Youth	6	50%
	Adult	4	33%
	Both	2	17%
Sport Type	Multiple sports (multi-sport clubs or project spanning various sport clubs)	6	50%
	Australian Rules football	2	17%
	School-based education targeting sport values generally	2	17%
	University sport	1	8%
	Soccer	1	8%
Intervention Target Group	Players/athletes	5	42%
	Mixed stakeholders (e.g., players, coaches, managers, and national body)	5	42%
	Coaches and support staff	2	17%
Intervention Type	Education-based programs and workshops	6	50%
	Collaborative coach learning discussions	1	8%
	Leadership/governance reform	1	8%
	Event-specific multi-component initiative	1	8%
	Club-level behavior change program (tiered accreditation system/policy)	1	8%
	Community partnership model	1	8%
	Inclusion model integrating sport/education/advocacy	1	8%
Outcome Data Source	Self-report only	9	75%
	Includes observation	3	25%
Outcome Assessment Timepoint	Concurrent or immediate post-intervention	9	75%
	Includes follow-up beyond intervention	3	25%

### Quality assessment

The methodological quality of each included study was appraised using the Mixed Methods Appraisal Tool (MMAT) (36). The MMAT is designed for systematic reviews that include qualitative, quantitative and mixed methods studies, and provides core criteria tailored to five study types: qualitative, randomized controlled trials, non-randomized studies, quantitative descriptive and mixed methods studies. Appraisal was conducted following the guidance set out in the MMAT user guide (37), with each study assessed against the criteria specific to its design.

Two reviewers (ZH and ST) independently assessed each study using the MMAT. Any discrepancies in scoring were resolved through discussion involving a third reviewer (JW). Rather than relying on a single composite score, the MMAT encourages reporting on each criterion to allow more nuanced interpretation of methodological strengths and limitations. This criterion-level approach is particularly important in reviews combining qualitative, quantitative and mixed methods studies, as the design-specific criteria are not directly commensurable across study types and should not be collapsed into a single quality ranking (36). Appraisal results were therefore summarized by criterion level and study design category, as presented in Supplementary Table S3.

## Results

**Figure 1:**
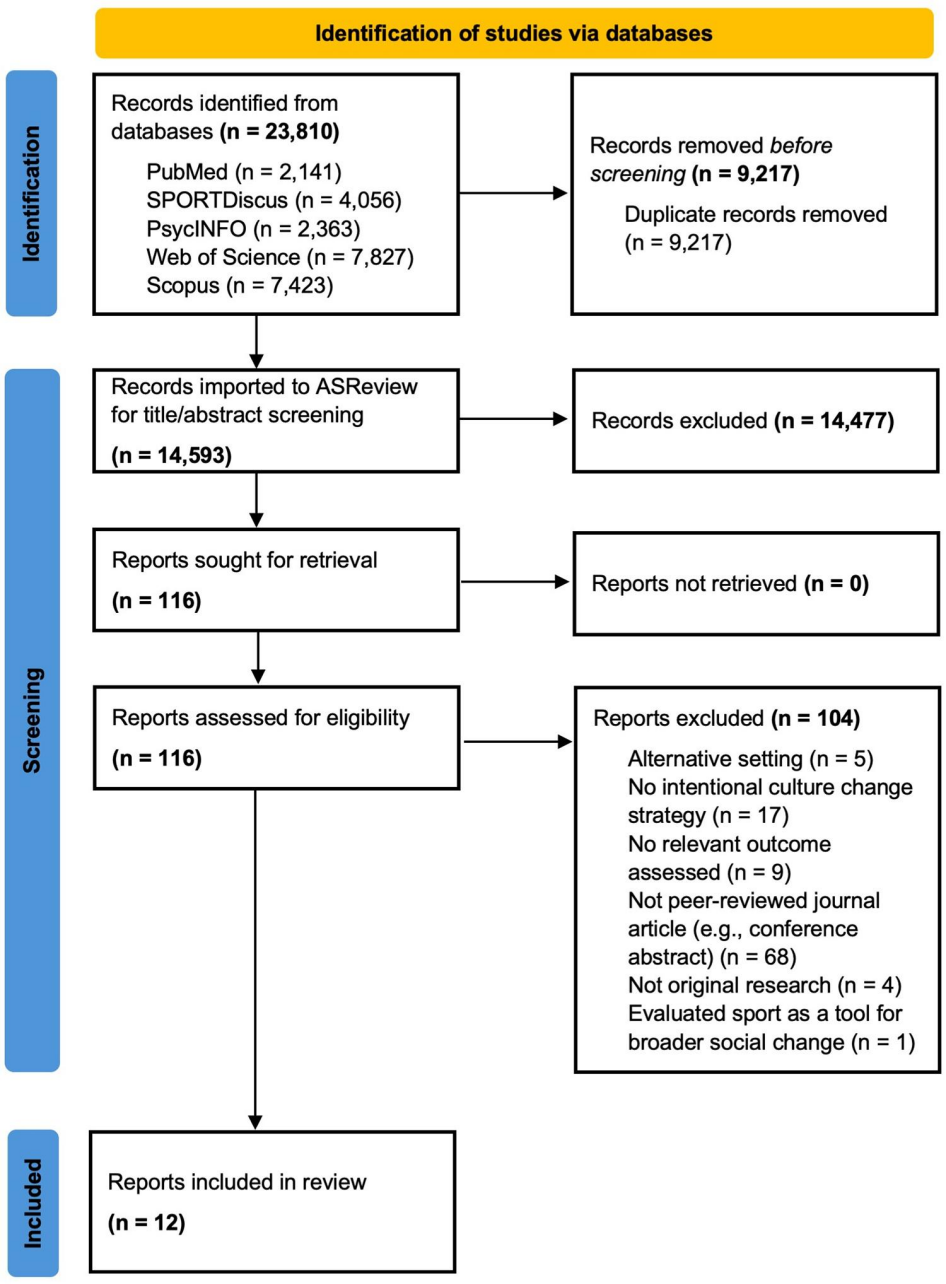
PRISMA 2020 flow diagram.

Twelve studies met the inclusion criteria for this review. Figure 1 presents the PRISMA flow diagram outlining the identification, screening and inclusion of studies. Studies varied widely in their design, sport setting and approach to culture change (Table 1). Most were published in the past decade, with studies from Australia comprising the largest proportion. Most studies used qualitative designs, and the interventions targeted both youth and adult sport. A notable proportion of studies were implemented in multi-sport community settings. Culture change strategies ranged from structured education programs and embedded coach learning cycles to governance reform and peer-led leadership initiatives. These efforts engaged a range of community sport stakeholders including players, coaches, administrators, parents and broader community members. Their focus ranged widely, aiming to address alcohol culture, inclusion of marginalized groups (e.g., Muslim women, people with disability, migrant-background youth), integrity, respectful behavior, and the values conveyed by adults in youth sport settings. While some interventions centered on shifting explicit practices and behaviors (e.g., drinking practices, inclusive coaching strategies), others sought more directly to engage deeper values, norms or relational dynamics within clubs and organizations. Across both types, however, the outcomes reported primarily reflect changes at the level of stated attitudes, knowledge, behavioral intentions or perceived shifts in club practices – surface-level indicators that, while consistent with culture change efforts, do not in themselves confirm that deeper organizational assumptions were reached or altered.

The following narrative synthesis groups included studies by their primary culture change focus. Three broad thematic clusters were identified: (i) *integrity and safety cultures*, encompassing initiatives targeting alcohol, performance-enhancing substance use, and sexual violence in sport settings; (ii) *inclusion and diversity*, covering efforts to improve belonging and participation of marginalized groups; and (iii) *adult conduct in youth sport and coach development*, including initiatives targeting coach and parent behavior in junior sport settings as well as broader coach development efforts aimed at shifting organizational learning culture. Within each theme, studies are described individually to preserve contextual detail. Key characteristics of each study, including design, sport setting, intervention features, outcome measurement and reported results, are further described in Table 2.

**Table 2:** Overview of included studies.

Reference & Country	Study design	Sport setting & target population	Culture change description	Outcome measurement	Reported results
Agnew & Pill (40) Australia	Qualitative evaluation	South Australian Southern Football League clubs (youth community football clubs in low socioeconomic areas in southern Adelaide) Junior footballers aged 14−17 years and club administrators	The South Connect Program is a youth development program that involved compulsory education sessions for junior footballers aged 14–17 on topics such as drugs and alcohol use, driver safety, depression, and respect for others.	Qualitative interviews	- Expressed belief that the program could “save lives” and prevent harm, though no measurable outcomes were reported. - Perceived improvements in responsible alcohol consumption among footballers. - Perceived increase in discussions about mental health issues, particularly depression. - Perceived cultural shift away from behaviors unfriendly to families and women and associated change in community reputation.
Barkoukis et al. (41) Greece	Quantitative; RCT (randomized at school level)	Coeducational high schools in urban and suburban cities in Northern Greece High school students	School-based anti-doping intervention, implemented through project-based learning over 20 contact hours as part of Health Education. The program was co-created with students and teachers and included multiple units on supplement and doping use.	Self-report questionnaires administered before and after the intervention	- Attitudes towards legal performance-enhancing substance use became less favorable in the intervention group compared to the control group. - No significant group x time interaction or main effect found for attitudes towards doping use. - No significant effects found for descriptive norms regarding the perceived prevalence of performance-enhancing substance or doping use. - Salience of doping use increased significantly in the intervention group post-intervention. - Health and ethics remained the top-ranked sport values pre- and post-intervention across both groups. - Doping became the most important threat to sports integrity in the intervention group's perception post-intervention.
Codella et al. (42) Italy	Quantitative; Pre-post evaluation	High schools in Italy High school students (15–18 years)	Lotta al Doping (Fight Against Doping) is a project that involved delivering standardized 2-hour seminars to high school students. The seminars were conducted by expert leaders and included PowerPoint presentations, scenario analysis, problem-solving, and small group discussions.	Anonymous online questionnaire (World Anti-Doping Agency Play True Quiz items).	- Significant improvement in knowledge about anti-doping rules and issues after seminars (13 out of 15 items correct; no control group).
Dyer & Sandford (46) England	Qualitative; Participatory research design	Rugby, rowing, cricket, boxing, tennis, golf, exercise, movement and dance (EMD) and bowls across the north of England Sport participants (disabled and non-disabled individuals), coaches, club managers, and National Governing Body representatives	The Mixed Ability model, developed by International Mixed Ability Sport, combines sport, education, and advocacy. It differs from other approaches by facilitating participation through reasonable adjustments rather than separate or adapted rules. It involves peer education by trainers with lived experience of disability and included 9 open days, 47 presentations, and implementation in multiple sports.	Qualitative tools including: Participant observation, in-depth interviews and focus groups, creative methods (e.g., photo elicitation, mind mapping) Multi-stakeholder workshops	- Perceived improvements in physical health and mental well-being. - Perceived shifts in club culture towards being more inclusive, with clubs better representing their local communities and coaches being more comfortable with diverse participants. - Increased awareness of barriers faced by different people to participating in sport and society more broadly. - Reported shifts in perceptions around dis/ability and social difference. - Non-disabled participants’ fears around communication difficulties with people they perceived as ‘different’ were alleviated. - The Mixed Ability model facilitated disabled people's participation in mainstream sports.
Ferris et al. (47) USA	Mixed-methods study with a waitlist control design	High school sports in the greater Boston area, including soccer, basketball, and tennis in four schools with varying socioeconomic and ethnic compositions. High school sports coaches	The Positive Coaching Alliance's “Double-Goal Coach” training program is a character-based coach education workshop. The program involves a one-to-two-hour interactive workshop at the beginning of the sport season, focusing on two main goals: scoreboard success and life lessons. It provides tools for character development in three domains: self, team, and game, and certifies coaches upon completion, with follow-up materials provided.	Open-ended survey questions administered at the end of the sport season	- Coaches reported increased self-reflection regarding their coaching behaviors following the program - Many coaches expressed intentions to change their coaching practices, with common changes including a more constructive, positive, and encouraging approach. - Coaches believed program contributed to valuing individuals and character development, fostering life skills beyond sport-specific outcomes. - Coaches observed improvements in team dynamics, including stronger peer support and leadership by team captains.
Hart (38) Australia	Qualitative; Case study	The “Northern Bats”, an Australian Rules football club in an outer suburb of Melbourne ranking in the lowest decile of national socioeconomic status Football club members and the local community (including a growing Islamic community)	The Good Sports Program is an intervention designed to change the drinking culture in sporting clubs. It involves a tiered accreditation system with criteria such as compliance with liquor laws, responsible service of alcohol training, provision of food and low-alcohol beverages, transport strategies to avoid drink driving, and promotion of the program. The program aims to reduce harmful drinking practices.	Semi-structured interviews with club and league officials Participant observation during a match day Field notes and narrative analysis of club dynamics	- Observed changes in the drinking culture within the clubrooms. - Increased diligence in serving alcohol and providing food and non-alcoholic beverages. - Perceived shift in norms towards responsible alcohol service and discouraging aggressive masculinity within the clubrooms. - Problematic drinking practices persisted in other settings, such as the car park.
Maxwell & Taylor (44) Australia	Qualitative; Case study	A community soccer club in the Canterbury local government area in South Western Sydney, Australia Muslim women and teenage girls, primarily of Middle Eastern (Arabic) background	A change in leadership occurred and two Muslim community leaders joined the board in 2005. The CSO introduced new equipment and uniforms sponsored by local partners, accepted the Hijab as part of the uniform, introduced culturally appropriate food, developed women-only training sessions, and appointed women as coaches. These efforts aimed to increase cultural awareness and inclusivity for Muslim women.	Semi-structured interviews (with participants, coaches, organizers) Focus groups Document analysis (club reports, media articles, public records)	- Participants reported perceived improvements in cooperation, trust, networks, and reciprocity through cultural accommodations. - The development of social networks and community partnerships enhanced trust and community belonging. - Improved cultural awareness and cooperative practices were seen as factors in engaging diverse religious and cultural groups. - The CSO's cultural profile changed from monocultural to multicultural, with increased participation of Muslim women.
Milistetd et al. (49) Brazil	Qualitative; Collaborative inquiry influenced by Appreciative Inquiry approach	Minas Tennis Club, a multisport club in Brazil Coaches, head coaches, and sport support staff within a sport club	The Learning in Action Project is a 24-month coach development initiative co-developed by researchers and sport club stakeholders. It was influenced by the Appreciative Inquiry approach and aimed to improve communication and collaboration among staff through sessions of learning discussions organized around 12 coaching topics.	Pre- and post-project interviews with staff Coach knowledge questionnaires Feedback forms during project	- Participants reported improved communication and collaboration among staff. - Project was perceived to facilitate exchanges and provide a rich learning environment. - Participants perceived improvements in understanding the club's broader context and a sense of community. - The project was not seen to substantially change the organization's culture.
Ramsden et al. (39) Australia	Qualitative evaluation	Uni Nationals, a bi-annual nation-wide university multi-sport competition University student sport team leaders	Multi-faceted culture-change intervention that includes leadership training and education for student team leaders, targeted harm-reduction messages, mocktail-making competitions, engagement of a Chef de Mission (elite athlete role model), and institutional support and expectation-setting from the university.	Semi-structured interviews (individual and group)	- Students were generally positive about the alcohol culture change interventions, but not all students were positive about the changes, indicating some resistance. - Leadership training component was perceived to have the greatest impact, empowering team leaders with confidence and skills to manage alcohol-related issues. - Targeted messages, mocktail events, and Chef de Mission were perceived as less effective by student leaders. - Culture change strategies improved students’ overall experience.
Schäfer-Pels et al. (43) Germany	Quantitative; Pre-post evaluation (no control group)	Organized sports in Germany Stakeholders in organized sports, including coaches, athletes, board members, and parents	A one-day workshop developed by the German Sport Youth Organization (dsj) aimed at preventing sexual violence in sports. It consists of two parts: raising awareness about sexual violence and learning prevention concepts. Participants develop practical steps for implementation and learn about handling suspected cases with counselling centers.	Standardized Theory of Planned Behavior-based questionnaires (before, immediately after, and 6 months after the intervention) Knowledge quizzes. Behavior self-reports and club-level change assessments.	- Positive short-term effects on attitudes toward sexual violence and intention to act against it. - Positive long-term effects on knowledge about sexual violence and culture of prevention in sports clubs. - More measures against sexual violence were implemented in sports clubs. - Subjective norms did not change over time.
Walters et al. (48) New Zealand	Mixed-methods design-based research approach; includes pre-post evaluation	Broader context of junior and youth sport in New Zealand, with specific involvement from organizations like North Harbour Rugby Union and integration into coaching materials for rugby Adults involved in youth sports: parents, coaches, club leaders, and sport administrators	Good Sports includes an adult education program to increase awareness of adult behavior impact, transformative learning with disorienting dilemmas, critical reflection, and dialogue, the Good Sports Spine (an evidence-based tool distinguishing between performance and development climates), a train-the-trainer model for community professionals, and infrastructure (developer network, public messaging, resources).	Surveys (pre/post-workshop) Semi-structured interviews Focus groups Observations Case studies (including developers, clubs, families, and organizations)	- Community modules positively influenced parents’ and coaches’ attitudes. Year one (96%) and year two (95%) respondents reported altered views on what comprises positive junior and youth sport experiences. - Nearly two-thirds (63%) considered the workshop's influence very strong. - Year one (86%) and year two (93%) respondents felt the module influenced their views on adult roles in junior and youth sport experiences. - Community modules heightened coaches’ and parents’ awareness of negative behaviors and initiated changes in thinking.
Young & Block (45) Australia	Mixed-methods - participatory action research with a pre-post evaluation design (quantitative component to be reported elsewhere)	Various community sport clubs Moreland and Hume, Melbourne, involving sports such as cricket, basketball, Australian rules football, soccer, futsal, badminton, and athletics. Young people from migrant backgrounds	Count Me In intervention includes employment of bicultural/bilingual community sport coordinators to link between families and sports clubs, development of resources for clubs (including to provide education around establishing an inclusive club environment) and families, and involvement of parent volunteers with children participating in clubs.	Surveys completed by children at start and end of program (most data not reported in this article) Semi-structured interviews and focus groups with children, parents, and sports club representatives	- Participants reported perceived improvements in physical and mental health and wellbeing due to increased activity and social interaction. - Parents noted an increase in their children's confidence. - Social benefits included new friendships and improved family relationships. - 60.4% of young people were still participating in organized sports at the end of the project.

CSO, community sport organization; RCT, randomized controlled trial.

### Integrity and safety cultures

Six studies addressed cultures relating to integrity and safety in sport, spanning alcohol, anti-doping, and sexual violence prevention. While the specific foci varied, these studies share a common concern with shifting the norms and practices that enable harmful behaviors within sport settings.

Hart (38) conducted a qualitative case study of an Australian Rules football club participating in the *Good Sports Program*, a tiered accreditation initiative promoting responsible alcohol service. Drawing on interviews with club officials and field observations, the study documented shifts in “official” norms of the clubroom, including responsible alcohol service, increased availability of non-alcoholic drinks, and efforts to include families and culturally diverse groups. However, problematic drinking behavior persisted in less-regulated areas outside the clubroom, where aggressive behavior continued among supporters. The study drew attention to the complexities of masculinity and place in influencing drinking norms, and the challenges of extending culture change beyond formal club settings.

Similarly, Ramsden et al. (39) qualitatively examined the impact of an alcohol culture change intervention implemented at Uni Nationals, a large inter-university multi-sport competition in Australia. The intervention included leadership training for team leaders, targeted messaging, mocktail events, and the appointment of an elite sporting role model as Chef de Mission. According to student team leaders through interviews, the leadership training – designed to build their confidence and decision-making capacity – was perceived to have the greatest impact. While other components were seen as less impactful individually, they contributed to an environment where sport was foregrounded over drinking. While authors noted that culture change can take time, some changes in alcohol culture were immediately recognized among Uni Nationals participants following this approach to change the environment and establish expectations.

Agnew and Pill (40) qualitatively assessed the *South Connect Program*, piloted in four South Australian football clubs. While broader in scope than a solely alcohol-focused initiative, encompassing education for junior players on mental health and respectful relationships alongside alcohol education, the program nonetheless centered on shifting the behavioral norms that commonly co-occur in community football club cultures. Delivered through workshops and community partnerships, interviews with club administrators before and after implementation indicated perceived shifts in relevant player behaviors and attitudes. Successful implementation was linked to strong local leadership and external support, though variability across clubs and reliance on volunteers posed challenges for sustainability.

Two studies evaluated school-based interventions aimed at promoting an anti-doping culture among adolescents. Barkoukis et al. (41) conducted an experimental study in Greece involving 218 high school students. The intervention, including health and morality perspectives among other components, was delivered over 20 contact hours using a project-based learning model. The intervention group showed significantly more negative attitudes towards legal performance-enhancing substance (PES) use post-intervention, and more strongly ranked doping as the greatest threat to sporting integrity. No significant change was observed in perceived prevalence or attitudes toward illegal PES use. Codella et al. (42) evaluated *Lotta al Doping* (*Fight Against Doping*), a large-scale anti-doping education campaign consisting of expert-led seminars, implemented across over 150 Italian high schools. Post-intervention, significant improvements were recorded in 13 of 15 knowledge items. Although the study lacked a control group, authors concluded that brief educational interventions can improve doping-related knowledge among adolescents on a large scale, supporting foundational culture change efforts in youth sport settings.

Schäfer-Pels et al. (43) evaluated a one-day sensitizing workshop developed by the German Sport Youth Organization, designed to prevent sexual violence in organized sports. The workshop was delivered to 137 stakeholders and assessed using the Theory of Planned Behavior framework. Short-term effects were positive across all measured factors: attitudes toward sexual violence, perceived behavioral control, intention to act against sexual violence, and knowledge all improved significantly, with large effect sizes. Long-term effects at six months were more mixed: only knowledge about sexual violence showed a sustained significant improvement, while attitudes, perceived behavioral control, and intention declined from their immediate post-workshop levels, though not to baseline. Importantly, the culture of prevention within participating clubs improved significantly at six months, and more protective measures against sexual violence had been implemented. Subjective norms did not change over time, which the authors attributed to this construct not being specifically targeted by the workshop. The authors concluded that single-session workshops can produce meaningful short-term sensitization and longer-term organizational behavior change, but that sustained attitudinal change likely requires more intensive or repeated intervention.

### Inclusion and diversity

Three studies examined efforts to create more inclusive community sport cultures for marginalized groups. Maxwell and Taylor (44) conducted a case study of a Sydney, Australia-based CSO that engaged Muslim women over a six-year period of cultural change and social capital development. Using interviews, focus groups and document analysis, the study found that cultural accommodations (e.g., allowing hijabs, women-only sessions), community outreach and leadership pathways supported trust-building and increased participation. The authors concluded that CSOs can build social capital and become more culturally inclusive by extending community networks, adapting practices and promoting cultural awareness.

Young and Block (45) conducted an evaluation of the *Count Me In* initiative in Melbourne, Australia, which used bicultural community support coordinators, local partnerships and resources for clubs and families to facilitate migrant-background youths’ participation in community sport. Their participatory action research engaged nearly 300 children and young people, mostly of Muslim background. By project end, 60 percent remained in organized sport. Findings of the qualitative evaluation suggested clubs that embraced cultural responsiveness retained participants more successfully. However, challenges included racism, cost barriers, and inconsistent willingness among clubs to accommodate cultural and religious needs, drawing attention to persistent systemic limitations to sustained inclusion.

Dyer and Sandford (46) examined the *Mixed Ability Sport Development* program in England, which promotes the participation of disabled people in mainstream sport via the Mixed Ability (MA) model. Through participatory qualitative methods, participants reported perceived improvements in physical health and mental wellbeing. Changes in club culture towards greater inclusivity, and challenges to dominant societal perceptions of disability, were also reported. The study concluded that while the MA model shows potential for facilitating authentic participation of disabled people in mainstream sports, ongoing education and support are necessary to ensure positive impacts and address cultural barriers.

### Adult conduct in youth sport, and coach development

Two studies evaluated approaches targeting adults in youth sport settings. Ferris et al. (47) examined high school coaches’ responses to the Positive Coaching Alliance's *Double-Goal Coach* workshop in the United States. The workshop is a one- to two-hour interactive training session designed to help coaches balance the pursuit of two goals: winning (goal one) and teaching skills through sport (goal two). Fifteen coaches provided open-ended feedback post-intervention. They reported intentions to change their behaviors after the workshop, becoming more constructive and positive in their approach. They also emphasized the importance of developing the “whole” athlete and fostering positive relationships between coaches, athletes and teammates. The study found that coaches valued the workshop content but suggested improvements such as more interactive sessions, sport-specific tailoring, and inclusion of athletes in the training.

Walters et al. (48) evaluated the *Good Sports* program in New Zealand (distinct from the alcohol-focused program of the same name), a three-year design-based research initiative using transformative learning workshops to engage over 4,000 parents, coaches and sport leaders. This culture change initiative was aimed at enhancing junior and youth sport experiences by influencing adult attitudes and behaviors. The workshops prompted reflection through disorienting dilemmas and discussions of adult influence in youth sport. Ninety-five percent of participants reported changed views on what constitutes a positive sport experience. Some organizations embedded *Good Sports* into policy and programming, including structural changes to competition formats to become more developmentally oriented. The collaborative approach between industry practitioners and academics was deemed essential to the project's success. Following the pilot, Good Sports was confirmed for national rollout by Sport New Zealand as the basis for its parent education approach.

Finally, Milistetd et al. (49) evaluated a 24-month collaborative coach development project in a Brazilian multisport club. Through iterative inquiry cycles involving over 100 coaches, the *Learning in Action* project led to reported improvements in internal communication and reflective practice. While it contributed to a more connected learning environment, it was concluded that the project “probably fell short…of significantly changing the culture of the organization”, with a follow-up assessment supporting that no significant structural changes had been embedded and that the new approach to coach development had not been sustained in daily routines.

### Quality assessment

Quality assessment results are presented in Supplementary Table S3. Overall, the qualitative studies were assessed as methodologically sound, with most meeting criteria related to appropriateness of approach, adequacy of data collection, and coherence between data, analysis and interpretation. Quantitative studies displayed greater variability in quality, with limitations particularly evident with respect to study design and completeness of outcome data, while mixed methods studies varied in the extent to which qualitative and quantitative components were meaningfully integrated.

## Discussion

This review examined how CSOs have attempted to change or develop their organizational culture, and to evaluate the outcomes of those efforts. Applying our inclusion criteria, only 12 peer-reviewed studies explicitly evaluating culture change in community sport could be identified. This represents a substantial gap between the prominence of culture change discourse in sport policy and practice, and the depth of empirical knowledge about whether and how such change occurs in community sport settings. The included studies, almost entirely published in the past decade, spanned a range of sports and contexts but were largely qualitative, small in scale and context-specific, which limits generalizability. Despite their heterogeneity, collectively the studies provide preliminary evidence that deliberate culture change in community sport can be associated with positive shifts in attitudes, behaviors and club practices. However, the extent and sustainability of these changes varied, reinforcing that transforming deeply embedded cultural norms in community sport settings is a complex and often long-term endeavor (50).

It is important to note that the outcomes reported across included studies predominantly reflect changes at the level of stated attitudes, knowledge, behavioral intentions or perceived club practices – what Schein (16) would characterize as artefact-level or espoused-values-level indicators – rather than evidence of shifts in the underlying assumptions that most durably constitute organizational culture. This does not mean the interventions failed to target culture in a meaningful sense; rather, it reflects the limitations in scope of the evaluation approaches employed, which were generally better suited to detecting immediate and proximal change than to assessing whether deeper cultural assumptions were genuinely altered. The fragmentation observed in how culture change is conceptualized, operationalized and evaluated mirrors earlier critiques of sport culture research more broadly (14), and resonates with parallel concerns in sport policy scholarship regarding conceptual inconsistency and weak alignment between frameworks and grassroots implementation (51).

A critical insight from this review is that culture change efforts in community sport tend to target specific issues aligned with broader social priorities in sport. Across included studies, culture change initiatives clustered around three areas: integrity and safety (including alcohol and anti-doping cultures), inclusion and diversity (e.g., increasing the participation and sense of belonging of women, ethnic minorities or people with a disability), and the values and conduct of adults in youth sport environments. Similar thematic emphases have been noted in wider sport policy and sociology literature, where community sport is increasingly positioned as a site for advancing social inclusion and health promotion (52). Collectively, these initiatives reflect an understanding of culture as multifaceted, encompassing observable practices (such as coaching behaviors or alcohol service policies) alongside less visible values and norms (such as attitudes towards diversity) underlying practice.

Approaches to intervention were accordingly diverse, ranging from structured education programs and workshops to embedded coach development cycles, governance reforms and peer-leadership initiatives. Notably, many initiatives combined individual-level learning (e.g., training for coaches, parents or club leaders) with organizational-level changes (e.g., introducing new codes of conduct, policies or messages within the club). This multi-level approach is consistent with organizational behavior theory suggesting that organizational goal achievement is supported by alignment of culture (e.g., shared values) with broader system structures and symbols (53). As Milistetd et al. (49) cautioned, translating individual learning into collective cultural change depends on alignment across leadership, organizational structures and everyday practice. In their case, learning at individual and group levels was not accompanied by sufficient structural change to support coach development, limiting the extent to which broader organizational culture shifted. Similar challenges have been observed in other multi-component health and inclusion initiatives in community sport, where policy reforms were introduced but required substantial external support to be enacted consistently at the club level, highlighting the limits of policy-led approaches in the absence of sustained implementation infrastructure (54). By contrast, some studies in our review did describe interventions that integrated education with changes to organizational practices or environments and subsequently reported shifts in norms or routines within clubs (for instance, clubs adopting new inclusive practices or alcohol management standards) (38, 46). The variation in intervention depth across included studies (from primarily educational or knowledge-based programs through to multi-component initiatives embedding structural and governance changes within clubs) itself reflects limited consensus about what culture change in community sport requires in practice.

Various studies reflected a tendency to engage a broad cross-section of stakeholders within the community sport environment. While earlier research on sport culture often emphasized top-down leadership as a means of strengthening shared values [e.g., Weese et al. (55)], the community sport initiatives in our review more commonly adopted participatory, bottom-up orientations. Coaches, parents, volunteers, club administrators, players and members of the broader community were involved as active contributors to change, rather than change being driven solely by those in formal leadership roles. These studies reported that sustained partnerships, community leadership and culturally responsive adaptation supported shifts in attitudes and relationships within clubs (44–46). This participatory ethos aligns with contemporary perspectives that stress collaboration and empowerment as foundations for social change, and work that has identified factors such as evidence-informed planning, the creation of mutually supportive environments, and sensitive facilitation that builds trust and local capacity as success factors for supporting inclusion (50). Similar conclusions have been drawn from research on inclusive participation models, which highlight the importance of flexibility and social connectedness in sustaining engagement (56).

Included studies described interventions that approached culture change through attention to both tangible behaviors and deeper values, reflecting broader theoretical distinctions between observable behaviors and the underlying meanings that sustain them. This dual focus aligns with established models of organizational culture, which conceptualize culture as operating across multiple levels, from visible artefacts and routines to less explicit norms and taken-for-granted assumptions (16). Within the reviewed interventions, some initiatives prioritized changes to explicit practices (such as alcohol service policies or coaching techniques) while others sought to engage more implicit norms, including how success, inclusion or appropriate conduct were understood within clubs. In some cases, these approaches were combined, pairing formal policy or procedural change with educational strategies designed to prompt reflection on attitudes and assumptions. For example, adopters of the New Zealand Good Sports program reported integrating values-based workshops for parents and coaches with organizational changes to policies, competition structures and delivery practices, with the explicit aim of reshaping adult norms and expectations in youth sport settings (48). However, the uneven transfer of resulting behavior change across settings [e.g., Hart (38)] can be understood through sociological accounts of how culture is reproduced in practice. From a theoretical perspective, such findings resonate with accounts of social life as structured by routine, embodied practices that reproduce cultural meanings over time, particularly in informal social spaces that are less amenable to formal regulation (57). Accordingly, while the findings of our review suggest that deliberate, multi-faceted efforts may disrupt entrenched habits, studies also noted pressures to revert to longstanding traditions (e.g., post-match drinking rituals) (38). This tendency highlights the resilience of informal norms and reinforces arguments that surface-level change is more readily achieved than shifts in deeply embedded cultural meanings, particularly where informal social spaces remain weakly regulated and symbolically powerful (16, 57, 58).

Across the included studies, outcomes were assessed using a wide variety of approaches, reflecting the heterogeneity of interventions. Most studies relied on self-reported measures – including interviews and surveys – collected over relatively short timeframes, with many concluding assessment at or shortly after the intervention. Where positive outcomes were reported, these typically reflected shifts in participants’ stated attitudes, knowledge or behavioral intentions, or perceived changes in club practices and relationships. This pattern is consistent with methodological observations in implementation science and organizational culture research that short-term attitudinal and self-report measures are among the most accessible but least conclusive indicators of change, tending to capture surface perceptions of what has shifted rather than deeper changes to organizational routines, practices and assumptions (59, 60). Few studies incorporated objective measures or formal indicators of sustained organizational-level change, and where organizational shifts were documented – such as embedding new practices – these were typically reported as perceived rather than independently verified. While longitudinal research designs capable of capturing whether organizational change efforts produce lasting effects do feature at encouraging levels in the broader nonprofit and community organization literature, the temporal dimension of organizational change remains underdeveloped (61). Indeed, the evidence reviewed herein is better positioned to speak to the immediate and perceived effects of culture change efforts than to whether those effects were durable or structural.

Relative to earlier reviews of sport organizational culture, this synthesis brings greater focus to change efforts within the specific setting of community sport. Previous reviews have shown that organizational culture research in sport has largely examined existing cultural characteristics and their associations with organizational features, most often within elite or university settings (14). In contrast, the studies included here reflect a growing, practice-oriented body of work concerned with influencing culture in grassroots sport settings. Further, whereas earlier studies provided limited attention to coaches’ or athletes’ experiences (14), reflecting a predominantly top-down viewpoint, the interventions in our review often involved participants beyond formal leadership – such as club members and parents – and measured outcomes at the level of club culture and member experiences. This shift broadens the empirical focus to include the perspectives of those who live the culture, aligning with calls for more inclusive, community-engaged research approaches in sport (50). Additionally, while the dominance of an ‘integration’ paradigm (seeing culture as unified and shared) has been identified in past sport studies, several studies in our review implicitly pointed to cultural variation within clubs. Rather than portraying culture as uniformly shared, these studies documented differences in how norms, values and practices were taken up across roles, settings or subgroups within the same organization [e.g., Hart (38); Walters et al. (48); Milistetd et al. (49)]. This suggests that community sport cultures are not always homogeneous, but may encompass multiple orientations and priorities that coexist within clubs, aligning with differentiation or fragmentation perspectives (14).

Consistent with broader organizational change scholarship, the reviewed studies suggest that change efforts seeking to alter core values in community sport are often slow and contested (50, 58, 62). Research in non-profit sport organizations suggests the relationship between culture and change is bidirectional: not only can existing culture constrain change efforts, but the introduction of change can itself trigger the formation of divisive subcultures that further impede progress (62). Long-standing informal rules, rituals and identities can generate inertia or resistance within volunteer-run organizations, particularly where clubs lack the authority, resources or mechanisms to mandate and monitor change, reiterating concerns that inclusive or reform-oriented policies may outpace the cultural readiness and operational capacity of some community sport settings (54, 63). Indeed, synthesizing accounts across multiple studies highlights that response to culture change in community sport is unlikely to be linear or uniform, and often unfolds amid negotiation between established traditions and reform-oriented aspirations (58).

At the same time, the review identified recurring facilitators of change in line with organizational change models (64). Committed local leadership, particularly when leaders acted not merely as administrators driving change but as visible champions who modelled desired norms, built relationships and networks that extended the reach of change efforts, and translated external aspirations into everyday club practice, was cited as enabling progress (44, 46, 64). External partnerships were also identified as a substantive facilitator. In these studies, partnerships with sporting bodies, health organizations, researchers or community organizations served distinct functions: providing the change initiative with credibility and legitimacy within the club context; transferring knowledge and skills that club personnel often lacked the expertise and capacity to develop independently; and supporting clubs to adapt their practices in culturally responsive ways to the communities they sought to include [e.g., Young and Block (45); Maxwell and Taylor (44)]. This multi-sector perspective and framing of change as part of broader movements aligns with public health perspectives that view community-level change as contingent on coordinated investments across organizations, rather than isolated initiatives (65).

Several studies further described the use of storytelling, emotional engagement and peer learning to prompt reflection and challenge entrenched assumptions, drawing on adult learning theories and social change principles. One program, for example, employed disorienting dilemmas – showing parents confronting videos of extreme sideline behavior – to spark critical reflection and empathy, leading to attitudinal shifts among parents and coaches (48). These results align with complementary evaluations of parent-education programs [e.g., Tamminen et al. (66); Sanders et al. (67)], contributing to the evidence that when adult stakeholders are equipped with shared values frameworks, youth sport cultures can become more supportive and inclusive. Taken together, these findings suggest that culture change efforts in community sport are supported through coordinated attention to organizational structures (policies, rules, resources), social factors (peer influence, leadership, community engagement) and individual factors (knowledge, attitudes). At the same time, they indicate that while progress can be made in creating safer, more inclusive and values-driven sport environments, such change is likely to be gradual where cultural norms are deeply ingrained and to require sustained effort over time (48, 50, 68).

A question worth considering in the context of our results is whether culture change efforts in community sport are primarily reactive – responding to a recognized problem – or proactive – seeking to build positive, inclusive and values-driven environments before problems arise. This distinction is well-established in the organizational change literature, which differentiates between reactive adaptation or re-creation in response to crisis or external pressure, and anticipatory change driven by strategic improvement or values-led design (69). Across the included studies, both orientations appeared evident, though such framing was rarely stated explicitly. Some initiatives were situated within broader sector-level responses to recognized harmful cultures – such as alcohol norms in community football (38, 39) and sexual violence in organized sport (43) – even where no specific antecedent within the participating organization was documented. The distinction matters as member responses to culture change may be guided by perceived fairness, necessity and values alignment. Where change is experienced as imposed or misaligned with core organizational values, compliance may be more superficial and less durable (70, 71). In sport, this dynamic has begun to receive empirical attention, with research on safeguarding in international sport federations finding that measures introduced in response to external pressures and rising stakeholder expectations often privilege initiatives with high symbolic value over comprehensive strategies with lasting impact – a pattern characterized as performative compliance (72). While the evidence across the studies reviewed does not permit direct comparison of outcomes across these motivational types, the distinction has practical relevance for how culture change programs are designed, framed and communicated to community sport stakeholders, and represents a direction for future research.

This review has several strengths. A comprehensive, systematic search was conducted across multiple databases, allowing inclusion of literature across disciplines. Screening was supported by an AI-assisted tool with rigorous human verification, and study quality was appraised using MMAT. Together, these approaches support the transparency and coherence of the synthesis. Some limitations should also be acknowledged. The review was restricted to English-language, peer-reviewed journal articles, and relevant evidence from grey literature or non-English contexts may have been missed. The included studies were predominantly drawn from Western, English-speaking countries, limiting the global generalizability of findings. The evidence base identified is also limited in important ways: many studies were small-scale, qualitative and of short duration, relied heavily on self-reported outcomes, and lacked long-term follow-up. Heterogeneity in how culture change was defined and assessed precluded quantitative synthesis and limits conclusions regarding the durability of observed changes. Finally, the requirement that study titles or abstracts explicitly use the term ‘culture’ – while allowing conceptual focus and supporting search practicalities, and consistent with prior systematic reviews in this area (14) – represents a meaningful limitation that warrants careful consideration. A substantial body of adjacent work in sport and organizational contexts examines functionally similar phenomena using related constructs such as organizational climate, norms, psychological safety and inclusion. By restricting to studies using the term ‘culture’, our review may have excluded meaningful work described under alternative terminology, and conclusions about the limitations of the evidence base should be interpreted with this in mind. Future reviews should consider broader or supplementary search strategies to capture this adjacent literature.

### Implications and future directions

The findings of this review have several implications for future research examining efforts to foster culture change in community sport. First, the fragmented and largely qualitative nature of the existing evidence base indicates a need for greater conceptual clarity and consistency in how organizational culture and culture change are defined and evaluated in community sport contexts. A foundational challenge here is that many existing approaches to measuring culture change may capture shifts in behavior without necessarily reaching the deeper values and assumptions that constitute culture itself (73). Developing shared theoretical grounding and more comparable approaches to assessment would strengthen the ability to interpret change across settings and over time. Future evaluations may also benefit from incorporating experience-based indicators to assess whether intended shifts in culture are meaningfully reflected in participants’ lived experiences [e.g., Brown et al. (74) Quality Sport Experience Framework for Youth].

Second, future research should prioritize longitudinal evaluation designs capable of detecting whether changes are sustained beyond the period of active intervention. The current evidence base is dominated by short-duration studies, with limited assessment of whether attitudinal or behavioral shifts endure or translate into lasting organizational change. Clearer and more consistent measurement of culture change outcomes – including the use of validated instruments and both individual and less commonly assessed organizational-level indicators – would substantially strengthen the comparability and cumulative knowledge-building potential of future research. Additionally, stronger theoretical frameworks that specify the mechanisms through which culture change occurs in community sport environments would help move the field beyond descriptive accounts toward explanatory models capable of guiding both intervention design and evaluation. Individual study findings point tentatively to conditions that appear to support positive outcomes – including committed local leadership, sustained external partnerships, and alignment between individual learning and organizational structures – though how these conditions interact and through what mechanisms they operate remains incompletely understood. That these conditions may appear in combination rather than in isolation suggests that culture change in community sport is better understood as a systems-level challenge than as a problem addressable through any single intervention component, and that future theoretical frameworks should attend to how these conditions interact.

For those designing and implementing culture change initiatives in community sport, the synthesis points to several practical considerations. Culture change initiatives are commonly embedded within broader social priorities and are contingent on local context, volunteer capacity and informal club norms. This highlights the importance of designing initiatives that allow for adaptation, provide sustained implementation support and align policy aspirations with the realities of grassroots delivery, where there is often a disconnect (58, 63). Indeed, the synthesis points to the value of participatory approaches that engage a wide range of stakeholders and attend simultaneously to organizational structures, social relationships and individual learning. However, persisting resistance, reversion to tradition and uneven change across clubs suggests that short-term or one-off interventions are unlikely to be sufficient. Supporting culture change in community sport therefore appears to require sustained investment, realistic expectations about the pace of change, and long-term alignment between policy, practice and community capacity.

This review demonstrates that intentional efforts to change organizational culture in community sport – across areas such as inclusion, integrity and adult behavior in youth sport – can be associated with meaningful shifts in attitudes, behaviors and practices. At the same time, it highlights that culture change in community sport settings is complex, uneven and often gradual, influenced by long-standing traditions, informal norms and resource constraints. While the evidence captured within our specific review criteria was limited, examples exist to support that community sport organizations can evolve toward safer, more inclusive and values-driven environments when change efforts are sustained, supported and contextually responsive. Strengthening this evidence base will require continued collaboration between researchers, policy-makers and practitioners, alongside more explicit theorization and evaluation of how culture change unfolds within the everyday realities of community sport – including through longitudinal designs, more consistent measurement frameworks, and stronger theoretical grounding for intervention development.
