# Supplementary material for: Changing organizational culture in community sport: a systematic review
Source: Front Sports Act Living. 2026 Jun 15;8:1852179. doi: 10.3389/fspor.2026.1852179 (PMC13310882; doi:10.3389/fspor.2026.1852179)
Supplement: Supplementary file 1 [file Table1.docx]

**Supplementary S1. Search Strategy**

| **PubMed**  ("sport*"[Title/Abstract] OR "Sports"[Mesh])  AND  ("community"[Title/Abstract] OR "youth"[Title/Abstract] OR "junior"[Title/Abstract] OR "school"[Title/Abstract] OR "grassroots"[Title/Abstract] OR "recreational"[Title/Abstract] OR "local"[Title/Abstract] OR "club"[Title/Abstract] OR "non-elite"[Title/Abstract] OR "amateur"[Title/Abstract] OR "social"[Title/Abstract] OR "casual"[Title/Abstract] OR "informal"[Title/Abstract] OR "voluntary"[Title/Abstract] OR "participat*"[Title/Abstract])  AND  ("cultur*"[Title/Abstract])  + English filter |
| --- |
| **SPORTDiscus**  (TI "sport*" OR AB "sport*" OR DE "AMATEUR sports" OR DE "AQUATIC sports" OR DE "BALL games" OR DE "INDIVIDUAL sports" OR DE "RACKET games" OR DE "RECREATIONAL sports" OR DE "SCHOOL sports" OR DE "TEAM sports")  AND  (TI ( "community" OR "youth" OR "junior" OR "school" OR "grassroots" OR "recreational" OR "local" OR "club" OR "non-elite" OR "amateur" OR "social" OR "casual" OR "informal" OR "voluntary" OR "participat*" ) OR AB ( "community" OR "youth" OR "junior" OR "school" OR "grassroots" OR "recreational" OR "local" OR "club" OR "non-elite" OR "amateur" OR "social" OR "casual" OR "informal" OR "voluntary" OR "participat*" ))  AND  (TI ( "cultur*" ) OR AB ( "cultur*" ))  + English, peer reviewed, academic database filters |
| **PsycINFO**  (TI sport* OR AB sport* OR DE "Sports" OR DE "Sport and Exercise Measures" OR DE "Adaptive Sports" OR DE "Athletes" OR DE "Athletic Participation" OR DE "Baseball" OR DE "Basketball" OR DE "Cycling" OR DE "Football" OR DE "High School Sports" OR DE "Judo" OR DE "Martial Arts" OR DE "Soccer" OR DE "Sports Coaching" OR DE "Swimming" OR DE "Tennis" OR DE "Weightlifting")  AND  (TI ( "community" OR "youth" OR "junior" OR "school" OR "grassroots" OR "recreational" OR "local" OR "club" OR "non-elite" OR "amateur" OR "social" OR "casual" OR "informal" OR "voluntary" OR "participat*" ) OR AB ( "community" OR "youth" OR "junior" OR "school" OR "grassroots" OR "recreational" OR "local" OR "club" OR "non-elite" OR "amateur" OR "social" OR "casual" OR "informal" OR "voluntary" OR "participat*" ))  AND  (TI ( "cultur*" ) OR AB ( "cultur*" ))  + English, academic database filters |
| **Web of Science**  (sport* (Title) or sport* (Abstract))  AND  ("community" OR "youth" OR "junior" OR "school" OR "grassroots" OR "recreational" OR "local" OR "club" OR "non-elite" OR "amateur" OR "social" OR "casual" OR "informal" OR "voluntary" OR "participat*" (Title) or "community" OR "youth" OR "junior" OR "school" OR "grassroots" OR "recreational" OR "local" OR "club" OR "non-elite" OR "amateur" OR "social" OR "casual" OR "informal" OR "voluntary" OR "participat*" (Abstract))  AND  ("cultur*" (Title) or "cultur*" (Abstract))  + English filter |
| **Scopus**  ( ( TITLE ( sport* ) OR ABS ( sport* ) ) )  AND  ( ( TITLE ( "community" OR "youth" OR "junior" OR "school" OR "grassroots" OR "recreational" OR "local" OR "club" OR "non-elite" OR "amateur" OR "social" OR "casual" OR "informal" OR "voluntary" OR "participat*" ) OR ABS ( "community" OR "youth" OR "junior" OR "school" OR "grassroots" OR "recreational" OR "local" OR "club" OR "non-elite" OR "amateur" OR "social" OR "casual" OR "informal" OR "voluntary" OR "participat*" ) ) )  AND  ( ( TITLE ( "cultur*" ) OR ABS ( "cultur*" ) ) )  + journal, article, English filters |
