# Supplementary material for: Changing organizational culture in community sport: a systematic review
Source: Front Sports Act Living. 2026 Jun 15;8:1852179. doi: 10.3389/fspor.2026.1852179 (PMC13310882; doi:10.3389/fspor.2026.1852179)
Supplement: Supplementary file 2 [file Table2.docx]

**Supplementary S2. ASReview Screening Procedure adapted from *SAFE* Procedure**

| **Phase 0. Consensus Screening**   - A purposive sample of 50 records was independently screened by five reviewers. - Discrepancies were resolved through discussion to ensure consistent application of inclusion criteria. - Final labels from this step were used as high-confidence training data to initialise the active learning model (Phase 2). |
| --- |
| **Phase 1. Screen a random set of training data**   - Reviewer (JW) screened a random sample of 1% of records (n = 146). - The proportion of relevant records in this sample (FRR_t = 0.014) was used to estimate the total number of relevant records in the dataset (RR_T = 204). - This estimate informed stopping criteria in subsequent phase (Phase 2). |
| **Phase 2. Apply active learning**   - Records were prioritised using active learning and screened in rank order by reviewer (JW). - The model was initialised using labels from the consensus screening phase and updated iteratively during screening. - Stopping criteria (all required):   - All key (known) relevant studies identified   - ≥ 2 × estimated number of relevant records screened (≥ 408)   - ≥ 10% of the dataset screened (≥ 1,459 records)   - No relevant records identified in the final 50 screened - Model configuration:   - *Query strategy:* Maximum   - *Feature extraction:* TF–IDF   - *Classifier:* Naïve Bayes   - *Balancing:* Dynamic resampling (double) |
| **Phase 3. Find more relevant records with a different model**   - Remaining unscreened records were re-ranked using an alternative model configuration. - Reviewer (JW) screened records in rank order. - Stopping criterion:   - No relevant records identified in the final 50 screened - Model configuration:   - *Query strategy*: Maximum   - *Feature extraction*: TF–IDF   - *Classifier*: Support Vector Machine   - *Balancing*: Balanced sample weighting |
| **Phase 4. Evaluate quality**   - Second reviewer (GT) screened previously excluded records ranked as most likely to be relevant. - The same model configuration as Phase 2 was used to prioritise records. - Stopping criterion:   - No relevant records identified in the final 50 screened |
