# Supplementary material for: Changing organizational culture in community sport: a systematic review
Source: Front Sports Act Living. 2026 Jun 15;8:1852179. doi: 10.3389/fspor.2026.1852179 (PMC13310882; doi:10.3389/fspor.2026.1852179)
Supplement: Supplementary file 3 [file Table3.docx]

**Supplementary Table S3. Quality Assessment Results**

| **Qualitative** | | | | | |
| --- | --- | --- | --- | --- | --- |
| **Study** | **1.1 Approach appropriate** | **1.2 Data collection adequate** | **1.3 Findings derived from data** | **1.4 Interpretation substantiated** | **1.5 Coherence across sources/analysis** |
| Agnew & Pill (2016) | Yes | Yes | Yes | No | Yes |
| Dyer & Sandford (2023) | Yes | Yes | Yes | Yes | Yes |
| Hart (2016) | Yes | Yes | Yes | Yes | Yes |
| Maxwell & Taylor (2010) | Yes | Yes | Yes | Yes | Yes |
| Milistetd et al. (2024) | Yes | Yes | Yes | Yes | Yes |
| Ramsden et al. (2021) | Yes | Yes | Yes | Yes | Yes |
| **Quantitative randomised controlled trial** | | | | | |
| **Study** | **2.1 Randomization appropriate** | **2.2 Groups comparable at baseline** | **2.3 Complete outcome data** | **2.4 Outcome assessors blinded** | **2.5 Adherence to assigned intervention** |
| Barkoukis et al. (2016) | No | No | Can’t tell | Can’t tell | Can’t tell |
| **Quantitative non-randomised** | | | | | |
| **Study** | **3.1 Participants representative** | **3.2 Measurements appropriate** | **3.3 Complete outcome data** | **3.4 Confounders accounted** | **3.5 Intervention /exposure as intended** |
| Codella et al. (2019) | Can’t tell | Yes | No | No | Yes |
| Schäfer-Pels et al. (2023) | Yes | Yes | No | Yes | Yes |
| **Mixed methods** | | | | | |
| **Study** | **5.1 Rationale for MM design** | **5.2 Integration of components** | **5.3 Interpretation of integration outputs** | **5.4 Address divergences/inconsistencies** | **5.5 Adherence to Qual + Quant quality criteria** |
| Ferris et al. (2015) | Yes | No | N/A | N/A | Can’t tell |
| Walters et al. (2022) | Yes | Yes | Yes | Yes | Yes |
| Young & Block (2023) | Yes | No | N/A | N/A | Yes |
| Note: Criteria labels above are concise; see MMAT (2018) manual for full wording and explanations. | | | | | |
